# Polymeric nanoparticles with a thermoresponsive shell loaded with fluorescent molecules allow for thermally enhanced fluorescence imaging and singlet oxygen generation†

**DOI:** 10.1039/d4na00687a

**Published:** 2025-01-09

**Authors:** Oksana Chepurna, Artem Yakovliev, Roman Ziniuk, Anna Grebinyk, Hao Xu, Olena A. Nikolaeva, Andrii I. Marynin, Liudmyla O. Vretik, Junle Qu, Tymish Y. Ohulchanskyy

**Affiliations:** a Key Laboratory of Optoelectronic Devices and Systems of Ministry of Education and Guangdong Province, College of Physics and Optoelectronic Engineering, Shenzhen University Shenzhen Guangdong P. R. China tyo@szu.edu.cn; b Department of Neurosurgery, Cedars-Sinai Medical Center Los Angeles CA USA; c Deutsches Elektronen-Synchrotron DESY Zeuthen Germany; d Taras Shevchenko National University of Kyiv Kyiv Ukraine; e National University of Food Technologies Kyiv Ukraine; f Engineering Research Center of Optical Instrument and System, Ministry of Education, Shanghai Key Lab of Modern Optical System, School of Optical-Electrical and Computer Engineering, University of Shanghai for Science and Technology Shanghai P. R. China

## Abstract

A thermosensitive polymeric nanoformulation (NF) was fabricated for thermally enhanced near-infrared (NIR) fluorescence imaging (FLI). It comprised core–shell nanoparticles (NPs) with a polystyrene core and a thermosensitive shell of a co-polymer of *N*-isopropylacrylamide and acrylamide [poly(NIPAM-*co*-AA)], which underwent a reversible conformational transition at 38–40 °C (corresponding to a lower critical solution temperature, LCST), leading to a reversible shrinkage of NPs from ∼250 nm to ∼140 nm for temperatures above LCST. The NIR dye 3782SL or photosensitizer HPPH were loaded to the NP shells. While the fluorescence of 3782SL and HPPH was quenched in water, it recovered in the NPs dispersion as a result of adsorption by NPs. Fluorescence for 3782SL and HPPH in NF increased when the temperature increased above LCST. Heating of HPPH-loaded NFs led to the elongation of the HPPH fluorescence lifetime and increased the generation of singlet oxygen (^1^O_2_). This occurred as a result of the NP shrinkage, corresponding shell compaction and NP aggregation, which hindered the internal conversion for photoexcited molecules adsorbed by NPs, and resulted in an increase in other deactivation pathways, namely fluorescence emission and intersystem crossing. The latter led to an increase in the triplet yield and, consequently, in singlet oxygen generation. Fluorescence microscopy revealed a 2–3-fold increase in the 3782SL or HPPH fluorescence signal from the NF-treated cells after they were heated up to 40 °C. Comparable results were obtained for the FLI of mice *in vivo*, after subcutaneous, intravenous, or intratumoral NF injections and localized heating by NIR (1.3 μm) laser irradiation. The developed NF holds immense potential for thermally enhanced FLI and photodynamic therapy.

## Abstract 2

Heating of core-thermoresponsive shell nanoparticles loaded with fluorescent molecules resulted in an increased fluorescence and singlet oxygen generation, which holds promise for thermally enhanced fluorescence bioimaging and photodynamic therapy.

## Graphical abstract

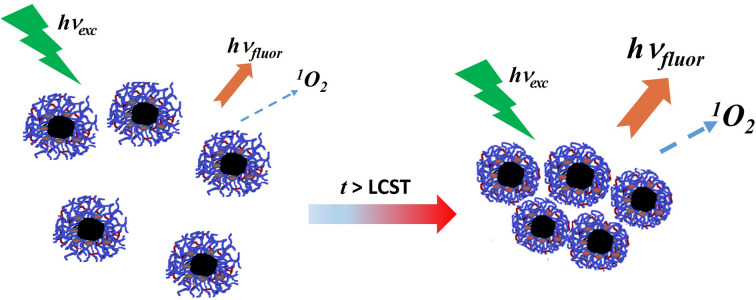

## Introduction

Fluorescence imaging (FLI) employing fluorescent contrast agents is a rapid, high-throughput and inexpensive optical imaging technology, which is particularly important for the biomedical field owing to its superior spatial and temporal resolution and the absence of ionizing radiation. FLI, which also includes fluorescence microscopy, allows for the visualization and probing of living cells and biological tissues *in vitro*, *ex vivo* and *in vivo*.^1,2^ FLI in the second near-infrared window of optical transparency (NIR-II, ∼1000–1350 nm) has recently emerged, demonstrating high contrast and sensitivity in comparison with the FLI of cells and tissues in the visible (VIS, 400–700 nm) and conventional near-infrared (NIR-I optical window, ∼700–950 nm) spectral regions. This recent progress is associated with the optical properties (*i.e.*, reduced light absorption and scattering) of tissues in the NIR-II and NIR-III (∼1500–1700 nm) windows, which lead to an enhanced contrast of the imaged biological structures at greater penetration depths compared with imaging in the VIS and NIR-I ranges.^3^ A low level of background tissue autofluorescence in the NIR-II, III windows (also referred to as short wave infrared range, SWIR) leads to an increased contrast for exogenous fluorescent (*i.e.*, luminescent, in broader sense) imaging probes.^4–6^ FLI in the SWIR range has recently been employed for the diagnosis of different diseases^7–10^ and also involved the use of SWIR luminescent inorganic nanomaterials, such as single-walled carbon nanotubes,^11–14^ quantum dots (QDs),^15–17^ and rare earth ion-doped nanocrystals.^5,18–20^ However, even though they show only low short-term toxicity, the inorganic luminescent nanomaterials raise critical safety concerns owing to their non-biodegradability and low extractability, leading to their retention in organisms and potential long-term toxic effects. Although the biological use of NIR-II small-molecule organic dyes has recently been demonstrated,^21,22^ a development of clinically relevant NIR-II dyes is considered to be a significant undertaking.^23^ The lack of biocompatible NIR-II luminescent imaging probes with high brightness and stability, along with low toxicity and suitable pharmacokinetics, can be considered a major bottleneck in the path towards clinical adoption of NIR-II FLI.^24^

With the development of nanotechnology over the last decades, the nanomedicine approach has emerged, bringing nanoparticles (NPs) to the biomedical field to improve existing disease treatments and introduce novel therapies. NPs can combine efficient drug loading with the controlled release and medical imaging modalities, allowing for the imaging and monitoring of NPs biodistributions and their delivery to the diseased tissues.^25^ Various formulations of NIR-I, II-emitting polymeric NPs have recently been developed as imaging probes for NIR-SWIR FLI, exploiting the characteristic biocompatibility and preparation feasibility, along with a great deal of flexibility in the tailoring of their chemical composition, size, biodegradability, morphology, and surface functionality.^25–27^ NIR fluorescent dye-loaded polymeric NPs have been reported in recent years, being formulated either by encapsulation of the fluorescent organic moieties in the process of the synthesis/preparation of the NPs,^28–32^ or by the adsorption of hydrophobic fluorescent molecules by pre-synthesized NPs in aqueous dispersions (“post-loading”).^5,33,34^ Due to their exceptional brightness, capacity to entrap different dyes, tunable sizes and available surface chemistry, these NPs are well recognized as prospective FLI agents and are increasingly used in various bioimaging applications.^28,35^ Recent advances in the development of NIR-SWIR FLI with fluorescent nanoprobes call for an introduction of NIR-SWIR-emitting theranostic nanoplatforms, capable of combining NIR-SWIR FLI with other imaging, therapeutic or sensing modalities.^36^

Stimuli-sensitive polymers are of longstanding interest to researchers and find broad biomedical applications due to the observed dynamic changes of their physicochemical properties in response to multiple external stimuli, such as pH, temperature, ionic strength, light, electrical and magnetic field.^37^ In particular, the thermosensitive polymer of poly(*N*-isopropylacrylamide) (PNIPAM) has attracted much attention, as the PNIPAM chains undergo an abrupt collapse to form hydrophobic nanospheres at and above a certain temperature (*i.e.*, lower critical solution temperature, LCST), showing a coil-globule volume transition; the LCST for PNIPAM in water is known to be ∼32 °C.^38,39^ PNIPAM-based NPs have been synthesized and applied in various fields, such as drug delivery and sensing.^40^ PNIPAM-grafted hydrophobic photoagents demonstrated the perfect integration of bioimaging and thermoresponsive properties.^38,41,42^ Thermosensitive NPs have been used to create hybrids with controlled fluorescence properties.^43^ Recently, polymer-embedded QDs nanospheres have been introduced; the combination of QDs and PNIPAM in NPs has been shown not only to improve the mechanical and chemical stability of QDs, but also to retain the properties of polymers and QDs; thus, revealing promising applications in the biomedical field, particularly as photoluminescent markers and drug delivery systems.^44^

We have recently reported on the thermoresponsive NPs for efficient NIR-SWIR imaging and imaging-guided drug delivery.^20,40^ The developed polymeric NPs have a polystyrene core and thermoresponsive shell of a co-polymer of *N*-isopropylacrylamide and acrylamide [poly(NIPAM-*co*-AA)], which can be loaded with near-infrared fluorescence dyes (NIRFDs) or co-loaded with NIRFD and photosensitizer (PS). It was found that the shift of the absorption of NIRFD to longer wavelengths strongly decreased the efficiency of the electronic excitation energy transfer between PS and NIRFD, leading to an increase in the efficiency of PDT with PS-NIRFD combination. It was also suggested by us that the use of NPs post-loaded with NIRFD emitting in the SWIR range can be beneficial for fluorescence imaging-guided PDT.^5,7,45^ However, the thermoresponsive behavior of the fluorescence from organic molecules post-loaded to the NPs was not studied or employed.

In this work, we report on thermosensitive nanoformulations (NFs) for thermally enhanced NIR FLI and PDT *in vitro* and *in vivo*. The developed NFs comprise core–shell NPs of ∼250 nm size, with a ∼30 nm polystyrene core and thermosensitive shell of the co-polymer of *N*-isopropylacrylamide and acrylamide [poly(NIPAM-*co*-AA)], which is loaded with NIR-SWIR fluorescent organic molecules: polymethine cyanine dye 3782SL fluorescing at ∼900–1200 nm or 2-[1-hexyloxyethyl]-2-devinyl pyropheophorbide-a, HPPH, which exhibits fluorescence in the ∼660–770 nm range and is a well-known photosensitizer for photodynamic therapy of cancer (PDT).^46,47^ With an increase in temperature of the NPs dispersion from ∼20 °C to ∼60 °C, the size of the NPs gradually changed from ∼250 nm to ∼140 nm. This change was shown to be reversible: the size increased back to ∼250 nm when the temperature was dropped back to 20 °C. The LCST of the NPs was determined to be ∼39 °C, which corresponds to the temperature at which the major change of the hydrodynamic diameter occurs. The fluorescence intensity for 3782SL and HPPH was found to rise when they were post-loaded to NPs. It also demonstrated a substantial rise with an increase in the temperature of the NPs dispersion above LCST, but was reduced back with the temperature decrease and corresponding increase in the NPs size. It is important to emphasize that the heating of NFs with HPPH above LCST was shown to result not only in fluorescence enhancement, but also in the elongation of the HPPH fluorescence lifetime and increased singlet oxygen (^1^O_2_) generation. After treatment of the cancer cells *in vitro* with NF, NIR-SWIR fluorescence microscopy of the treated cells revealed not only cellular internalization of NF, but also a ∼2–3 fold increase in the fluorescence of 3782SL or HPPH from the cells after they were heated up to 40 °C. Comparable results were obtained for NIR-SWIR fluorescence imaging *in vivo*: the NIR-SWIR signal from the NF containing the 3782SL dye increased by ∼2 times after the subcutaneous injection site was heated externally using irradiation by the 1.3 μm laser diode. Moreover, a similar enhancement was observed in the NIR-SWIR fluorescence imaging of mice intravenously injected with 3782SL-loaded NF: blood vessels with circulating NF were highlighted by the enhanced NIR-SWIR fluorescence in the locations heated by the irradiation from the 1.3 μm laser diode. Finally, an enhancement in the NIR fluorescence from HPPH *in vivo* was observed after the mouse subcutaneous tumor was injected with NF/HPPH, followed by laser irradiation.

## Results and discussion

### Preparation and morphological characterization of the thermoresponsive core–shell NPs

**Fig. 1:**
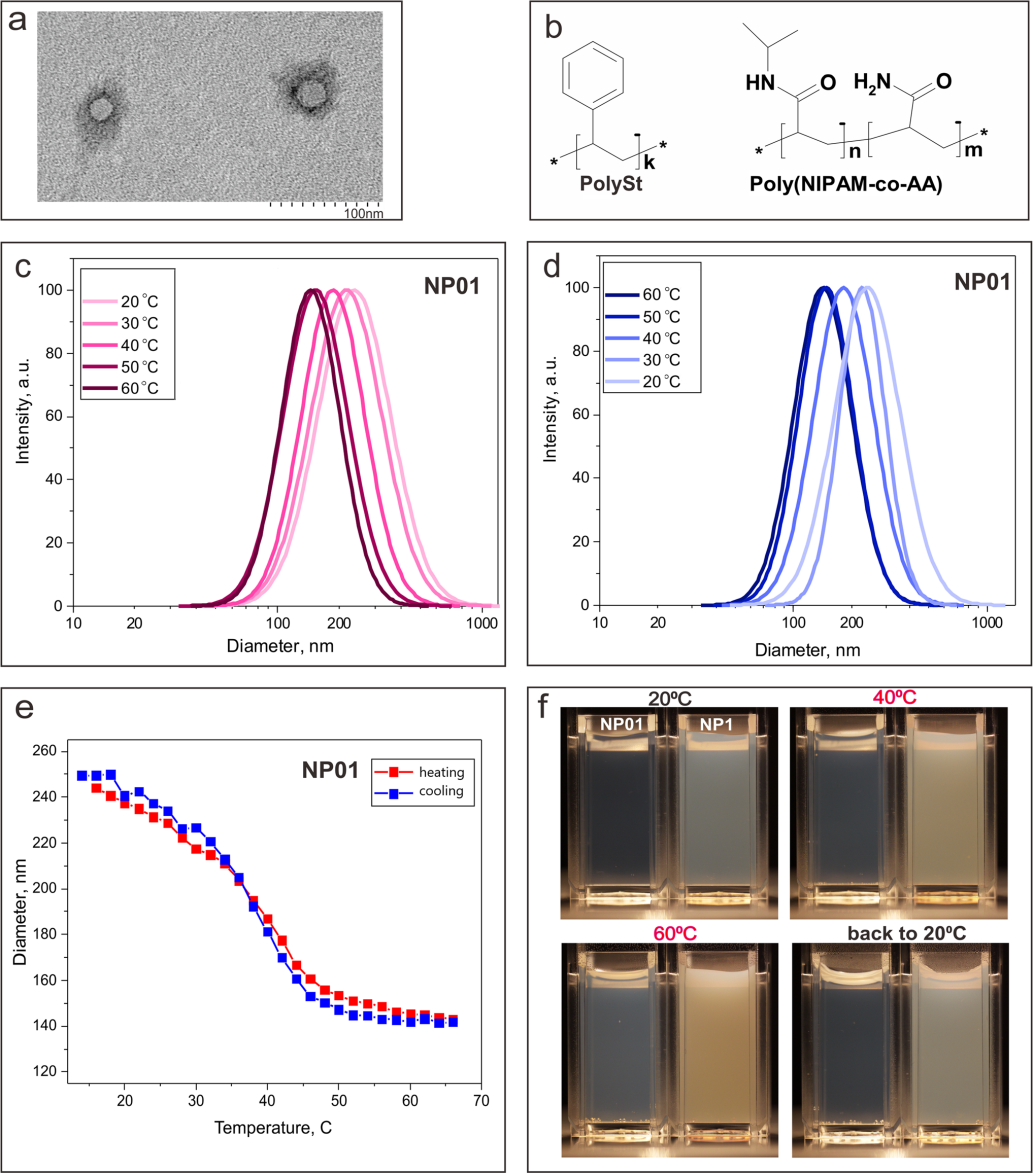
(a) TEM image of the polySt-poly(NIPAM-*co*-AA) core–shell NPs; (b) chemical structures of polymers comprising NPs; (c) DLS results on the change of the NPs size distribution with the heating of the NPs water dispersion (0.1 w/v); (d) DLS results on the change of the NPs size distribution with cooling NPs water dispersion (0.1 w/v); (e) DLS results on the change of the NPs size with the heating and cooling of the NPs water dispersion (0.1 w/v); (f) photographic images of the NP01 and NP1 water dispersions (concentration of NPs 0.1% w/v and 1% w/v, correspondingly) at 20 °C, heated to 40 °C and 60 °C, and then cooled back to 20 °C.

In the synthesized NPs, the core comprises PolySt, while the shell consists of a poly(NIPAM-*co*-AA) copolymer (Fig. 1b). The core–shell structure of the NPs is revealed in the TEM image (Fig. 1a and S1†). At the same time, while the ∼30 nm polySt core is clearly observed, the shell thickness cannot be revealed by TEM image, as the drying of the NPs dispersion on the TEM grid is known to result in shrinkage of the poly(NIPAM-*co*-AA) shell.^40^ Upon heating in water solution, the PNIPAM polymer NPs undergo a conformational transition, expelling water molecules and shrinking at temperatures above the LCST.^48^ On the other hand, while LCST for the PNIPAM homopolymer is ∼32 °C, copolymerization of the monomer NIPAM with more hydrophilic monomers, (*e.g.*, acrylamide, AA), at different monomer ratios provides an opportunity to precisely tune the LCST up to physiological temperatures and higher.^38,40,49^

DLS measurements allowed us to evaluate changes in the NPs size, which are associated with conformational changes of the copolymer shell with temperature change (Fig. 1c–e). While the average hydrodynamic diameter of polySt-poly(NIPAM-*co*-AA) NPs in dispersion at room temperature (∼20 °C) was determined to be ∼250 nm, it decreased with heating and the NPs shrunk down to ∼140 nm at the highest of the assessed temperatures (∼58–68 °C). On the other hand, the NPs swelled up during the course of the dispersion cooling down, returning to the initial size of ∼250 nm at ∼20 °C (Fig. 1e). It should be noted that the NPs size distribution does not change significantly during the heating and cooling processes (Fig. 1c and d). The LCST of the developed NPs was determined to be 39 °C, which is close to that for similar NPs reported by us earlier.^40^ This suggests that these NPs can be more colloidally stable at physiological temperature (*i.e.*, ∼37 °C) in comparison with pure PNIPAM nanoparticles, which are known to aggregate at temperatures higher than LCST for PNIPAM (∼32 °C).^50^ While experimenting with heating and cooling of NPs dispersions of different concentrations in water (0.1%, NP01, and 1%, NP1), we have noticed slightly different behavior for the NP01 and NP1 samples, which can be seen in the photographic images presented in Fig. 1f. After heating at the hotplate to 40 °C and then 60 °C, the NP01 dispersion becomes visibly more opaque. However, after cooling back to 20 °C, its initial transparent appearance was nearly restored. This observation is evidently associated with the shrinking of the NPs at higher temperatures. It should be noted that at temperatures below LCST, PNIPAM is engorged with water molecules and has a refractive index that is comparable to water. Thus, the light scattering is not as significant. In contrast, at temperatures above LCST, the water molecules are expelled from the polymer, which leads to a noticeable change of its refractive index and increase in light scattering.^51^ Thus, light scattering by the NP01 dispersion rises even if the size of the NPs decreases. At the same time, one can see that the NPs sample with higher concentration, NP1, remained significantly more opaque after cooling back to 20 °C than it was before heating (Fig. 1f). This suggests that NPs at higher concentrations do form aggregates at temperatures above LCST, and these aggregates can at least partially remain after the dispersion is cooled back to lower temperatures. This phenomenon is not as clearly pronounced for lower concentrations. It is not as visible at lower concentrations, and the DLS measurements do not reveal any aggregate formation for the NP01 dispersion after cooling back to 20 °C (Fig. 1c, d and S2†).

### Spectroscopy characterization, NIR-SWIR cell imaging and *in vitro* toxicity of NF with NIRFD

**Fig. 2:**
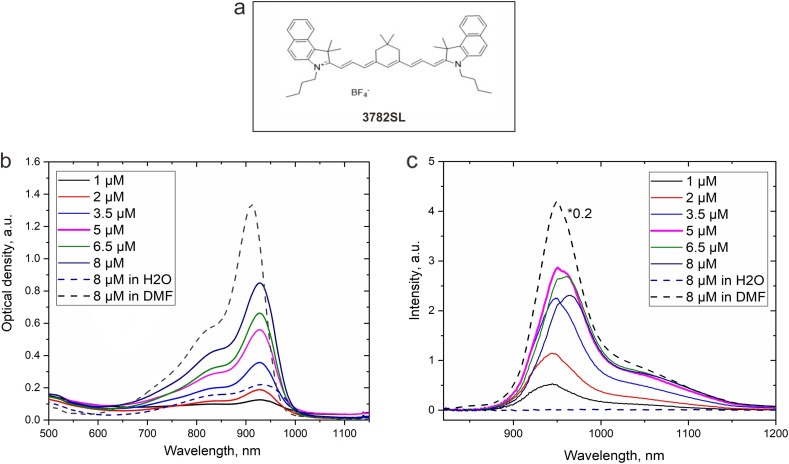
(a): Chemical structure of the NIR fluorescent dye 3782SL; (b and c): absorption (b) and fluorescence (c) spectra of the 3782SL dye in water, DMF, and in the NPs water dispersion (NP01D nanoformulation).

Following the characterization of the temperature-dependent behavior of NPs, we proceed to the preparation and characterization of NF loaded with fluorescent molecules. NIR fluorescent NF were prepared by post-loading of 3782SL dye (Fig. 2a) into polySt-poly (NIPAM-*co*-AA) core–shell NPs. The 3782SL dye from a stock solution (1 μM in DMF) was added to 2 mL of NP1 at different molar concentrations (1; 2; 3.5; 5; 6.5; 8 μM) and left in the dark overnight. The next morning, absorption and fluorescence spectroscopy measurements were performed. As shown in Fig. 2c, the dye is virtually non-fluorescent when its stock DMF solution is suspended in water, while it manifests strong fluorescence in DMF solution. At the same time, the absorption of the dye in water is seen to be much weaker than that of the dye DMF solution (Fig. 2b, curves for the 8 μM dye concentration in DMF and water). This difference in absorption and fluorescence is apparently associated with the aggregation of 3782SL in water. While suspended from the DMF stock solution in the NPs water dispersion (NP01), the dye partly restores its fluorescence through post-loading into the NPs (*i.e.*, *via* adsorption by the NPs shell), although its fluorescence intensity remains much lower than that of the DMF solution of 3782SL (with the same concentration). The concentration of 3782SL loaded into the NPs was optimized to allow for the highest fluorescence signal. As one can see in Fig. 2c, at a constant NPs concentration (0.1% w/v), the 3782SL fluorescence intensity rises almost linearly with the dye concentration until 5 μM. However, upon an increase of the dye concentration to 6.5 μM, there is not a corresponding increase in the fluorescence intensity and it even exhibits a slight decrease. This decrease became evident upon further increasing the dye concentration. We suggest that at high dye concentrations with the NPs, the dye molecules occupy all of the available post-loading sites in the NPs shell and begin sticking to the NPs surface, interacting with each other (*e.g.*, aggregating) and causing fluorescence quenching, which results in a shift of its peak towards longer wavelengths (Fig. 2c). This effect is similar to that observed when molecular fluorescent probes interact with DNA, aggregating on the DNA molecules at high dye:DNA ratios.^52^ To avoid fluorescence quenching, the NIRFD concentration of 5 μM was used in further experiments with NF containing NIRFD.

**Fig. 3:**
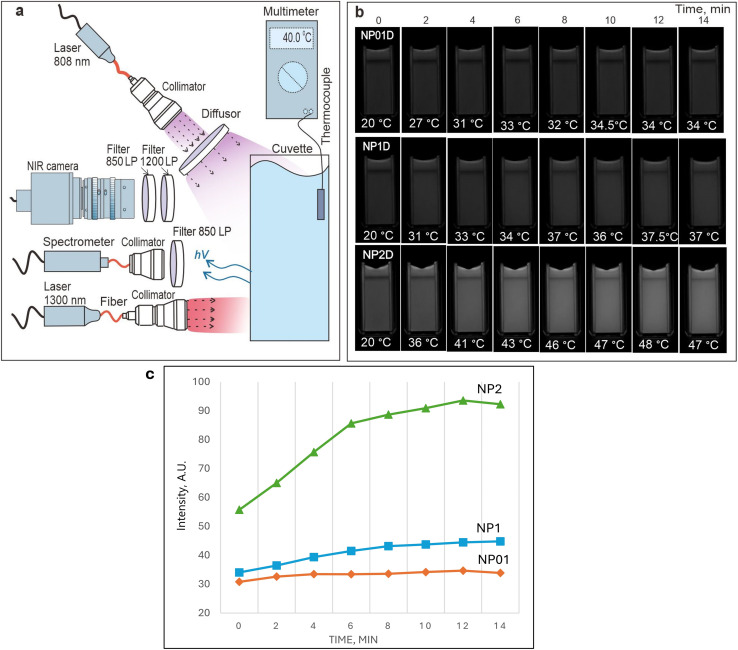
(a) Scheme of the setup employed for evaluating the changes in the NIR-SWIR fluorescence from NFs heated by the 1300 nm laser; (b) NIR-SWIR fluorescence images of NP01D; NP1D; NP2D nanoformulations in the course of heating by the 1300 nm laser; (c) dependence of the NIR-SWIR fluorescence intensities on the time of irradiation.

After obtaining NP01D and NP1D nanoformulations, the sensitivity of their NIR fluorescence to temperature was evaluated. Aiming for the preplanned *in vivo* experiments with localized heating of the animal body, we have employed a 1300 nm laser to heat NF through water absorption, as NPs and 3782SL dye do not absorb light at this wavelength but water does. A scheme of the experimental setup is shown in Fig. 3a; it involves: (1) a cuvette with the NF sample, which is irradiated by a defocused 808 nm laser to excite the fluorescence and by a 1300 nm laser to heat the NF (the cuvette also contains an immersed thermocouple to measure the temperature); (2) a NIR-SWIR camera to image the NF fluorescence from the NF (the camera is equipped with long and short passes optical filters to cut off the laser light scattering); (3) a NIR-SWIR spectrometer to measure the NIRFD fluorescence spectra. We observed that when the NP01D and NP1D were continuously irradiated by the 1300 nm laser, the temperature of the NF samples rose, with a higher temperature increase for the more concentrated NF. This trend became obvious when we explored the behavior of the NIRFD fluorescence in an even more concentrated NPs water dispersion (2% w/v, NP2D). It should be noted that the NP01D, NP1D and NP2D NFs contained the same concentration of 3782SL dye (5 μM). Despite this, the fluorescence signal from NP01D, NP1D and NP2D is noticeably different, and the signal was notably stronger for the more concentrated NFs. It is worth noting that the spectral position and shape of the fluorescence band from NIRFD did not change during or as a result of heating NP01D, NP1D and NP2D (Fig. S3†). These results suggest that the fluorescence of the NIRFD post-loaded to NPs can be enhanced both by an increase in temperature of the NPs dispersion and in the NPs concentration. As discussed above, the NPs aggregate more readily at the higher concentration, and this can be a reason for the stronger fluorescence and higher temperature rise.

**Fig. 4:**
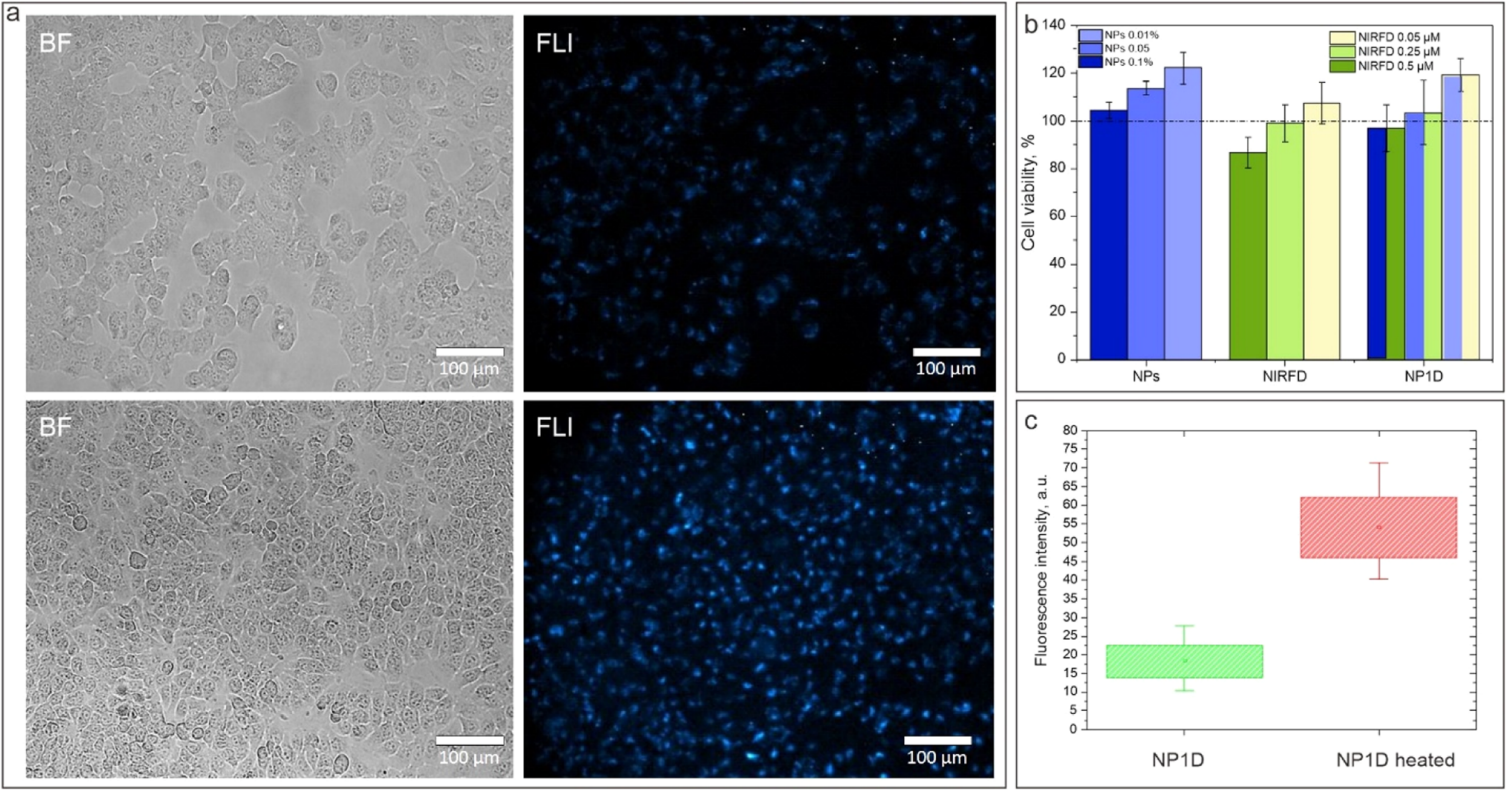
(a) Transmission (left column) and NIR-SWIR fluorescence (right column) microscopy images of LLC cells treated with NP1D, before (top) and after (bottom) heating; (b) LLC cell viability after 24 h treatment with blank NPs, free NIRFD and NP1D; (c) histogram of the fluorescence signal from cells treated with NP1D, before and after heating.

To assess the bioapplicability of the developed NFs, the cell viability was assessed for cultured cells treated with NIRFD-containing NF, “blank” NPs and free NIRFD. While the free NIRFD exhibited slight toxicity towards LLC cells at 5 μM concentration, it was not observed once the dye was post-loaded in the NFs. The NFs effect on the cell viability was similar to that for the “blank” NPs at the studied concentrations (Fig. 4b). The obtained data reveal the negligible toxicity of the developed NF containing 5 μM of NIRFD towards the LLC cells. Thus, after spectral characterization and cell viability assay, the NP1D, which contained 1% w/v of NPs post-loaded with 5 μM, was chosen for *in vitro* and *in vivo* experiments.

The *in vitro* cell uptake for NF containing NIRFD was assessed using LLC cells. The transmission and NIR-SWIR fluorescence microscopy images clearly reveal cell labeling with NIRFD after incubation of cells with NP1D NF (Fig. 4a). Furthermore, when a dish with the cells was placed on a hotplate that was heated to 40 °C for 2 min, and then immediately returned to the microscope stage and imaged, the NIR-SWIR fluorescence signal from the cells was found to significantly (2–3 fold) increase, suggesting that NP1D NF within the cells retains its shrinking/aggregating behavior that causes the increase of the fluorescence signal from the post-loaded fluorophores.

It should be noted that the NIR-SWIR fluorescence microscopy imaging of LLC cells treated with free NIRFD was also performed as a control experiment (see ESI, Fig. S6†). While the NIR-SWIR fluorescence signal was also detected/imaged after incubation of the cells in the cell medium with free NIRFD, the free NIRFD-treated cells did not reveal any difference in the level of NIR-SWIR fluorescence signal from the cells before and after heating, in contrast to the cells treated with NP1D.

### Spectroscopy characterization, cell imaging and *in vitro* toxicity of NF with PS

**Fig. 5:**
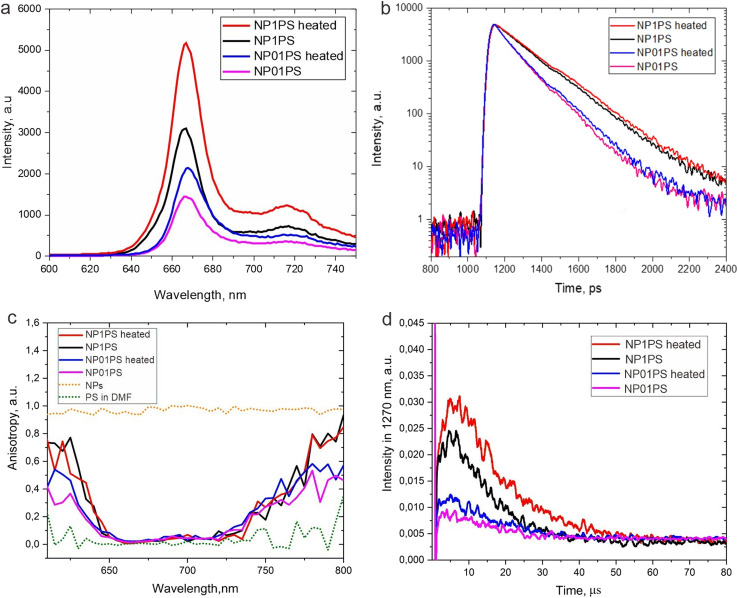
(a) Fluorescence spectra of NP01PS and NP1PS nanoformulations with and without heating. (b) HPPH fluorescence decays for unheated and heated NFs of NP01PS and NP1PS; (c) HPPH fluorescence anisotropy spectra for NFs with and without heating, free PS in the organic solution and NPs without post-loaded HPPH. NFs; (d) decays of phosphorescence (detected at 1270 nm) of singlet oxygen sensitized by NP01PS and NP1PS with and without heating.

Being intrigued by the temperature-dependent changes in fluorescence from 3782SL post-loaded into NPs, we decided to assess the thermoresponsive behavior of another fluorophore adsorbed by the nanoparticle shell. We have previously reported on the post-loading of photosensitizer HPPH (2-[1-hexyloxyethyl]-2-devinyl pyropheophorbide-a) to polySt-poly(NIPAM-*co*-AA) core–shell NPs, and found that post-loading (adsorption of HPPH) by the NPs shell precludes aggregation of its hydrophobic molecules in water, preventing fluorescence quenching and reduction of singlet oxygen generation.^40^ In this regard, we opted for HPPH as a molecule with fluorescence whose thermoresponsive behavior is to be explored, when HPPH is loaded to the polySt-poly(NIPAM-*co*-AA) core–shell NPs. It is important to keep in mind that HPPH is also capable of singlet oxygen generation, which is employed in PDT;^46,47^ that is why the thermoresponsive behavior of singlet oxygen generation also can be explored. After the post-loading of HPPH to NPs, as described in Materials and methods section, we studied the change of its photophysical properties in the resulting NF at two different concentrations and in non-heated (room temperature) and heated (∼40 °C) states. The heating was performed by irradiation from a 1300 nm laser, and the temperature in the cuvette was directly measured by the immersed thermocouple. It was observed that heating NF with concentrations of NPs of 0.1% and 1% (named NP01PS and NP1PS, respectively) significantly increases the HPPH fluorescence intensity, with a larger increase observed for a higher concentration of NPs (Fig. 5a). It is also important to note that the larger increase in the HPPH fluorescence intensity correlates with an increase in the fluorescence lifetime. Fig. 5b shows that the HPPH fluorescence lifetime is longer for NF with higher NPs concentration (N1PS). Additionally, it appears to become even longer after the heating of NF, although the difference is rather small. The same trend is also observed for NP with lower concentrations of NPs (N01PS), allowing us to suggest that the intensity and lifetime of the HPPH fluorescence increases at the expense of the greater rigidity/viscosity of the NPs matrix at higher concentration and temperature. However, it should be noted that the statistical significance of the change in the HPPH fluorescence decays between the heated and unheated NF has not been evaluated, so any conclusion is tentative, and this will be the subject of future studies. It can be only assumed that the rate of internal conversion in the photoexcited PS molecule is reduced when it is fixed within a shrunken shell (especially at higher concentration of NPs) in comparison to that of the PS molecule loaded in the swollen shell and at lower concentration. If this is the case, the rate of other singlet excited-state deactivation processes, such as fluorescence emission and/or intersystem crossing, would then increase. It is worth noting that organic molecules can be virtually nonfluorescent in solution, but “switch on” the fluorescence signal in viscous or cold environments, or by binding to oligonucleotides or other macromolecules.^53–57^ To ensure that HPPH molecules are tightly associated with the nanoparticle shell, we performed fluorescence anisotropy measurements, which can prove that the fluorophore is bound with NPs.^58,59^Fig. 5c reveals that the free HPPH solution in DMF shows negligible fluorescence anisotropy. In contrast, HPPH loaded to NPs exhibit high fluorescence anisotropy, although no difference between the heated and unheated dispersions of different concentration could be seen. It should be noted that the anisotropy value, which is experimentally measured by a spectrofluorometer, is sensitive to the scattering of the excitation light. As a result, much higher scattering by the dispersion of the NPs results in a different baseline for the anisotropy measurements than that for the molecular HPPH solution. This can be illustrated with a fluorescence anisotropy measurements for the “blank” (non-fluorescent, without HPPH) NPs dispersion, which is shown in Fig. 5c as a dotted yellow line; it can be considered as the baseline for the anisotropy measurements of the fluorescence from the NPs.^58,59^

As the fluorescence anisotropy measurements confirmed the tight entrapment of the HPPH molecules in the NPs shell, we further assessed whether this entrapment causes not only fluorescence enhancement, but also increases the singlet oxygen generation. In fact, hindering the internal conversion for photoexcited molecules adsorbed by NPs should lead to an increase in other deactivation pathways, *i.e.*, fluorescence emission and intersystem crossing. The latter, in turn, would lead to the increase in singlet oxygen generation.^57^Fig. 5d illustrates the determination of singlet oxygen produced by HPPH in NP01PS and NP1PS nanoformulations. The singlet oxygen generation was assessed by the time-resolved measurements of ^1^O_2_ phosphorescence peaked at ∼1270 nm. This method allows for direct evaluation of the amount of generated ^1^O_2_, which is proportional to the area under the ^1^O_2_ phosphorescence decay curves.^40,41,60^

As one can see in Fig. 5d, the heated NP01PS and NP1PS do produce more singlet oxygen than the unheated formulations NP01PS and NP1PS, which correlates with the difference in the HPPH fluorescence intensity from these two nanoformulations. Moreover, it should be noted that similar to the HPPH fluorescence intensity, the ^1^O_2_ emission signal is significantly higher for the NF with a higher concentration of nanoparticles (NP1PS) than for the NF with a lower NPs concentration (NP01PS), even though the HPPH concentration was the same. Interestingly, on comparing Fig. 5a and d, one can see that the intensities of the HPPH fluorescence and singlet oxygen phosphorescence similarly increase when the NPs concentration or temperature increase. This observation supports the assumption that both fluorescence and singlet oxygen production by HPPH molecules adsorbed by NPs increase as a result of the temperature-induced NPs shrinkage, corresponding to shell compaction and NPs aggregation, which leads to hindering the internal conversion in photoexcited PS molecules loaded to the NPs shell.

**Fig. 6:**
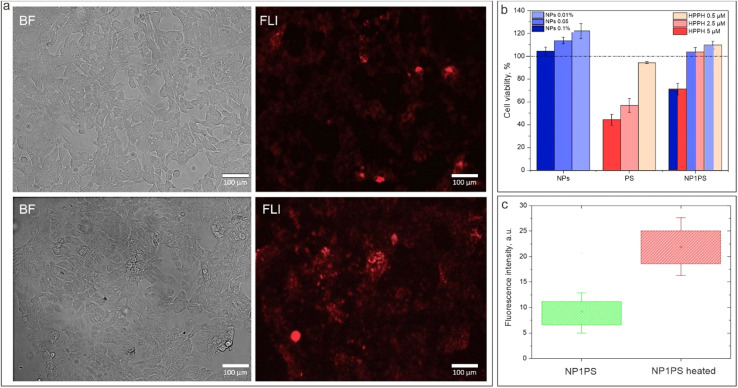
(a) Transmission and fluorescence microscopy images of cultured live cells treated with NP1PS before (top) and after (bottom) heating; (b) viability of cultured cells after treatment with NPs, free HPPH and NP1PS for 24 h; (c) histogram of the fluorescence intensity from images of cells treated with NP1PS before and after heating.

As a next step of the studies, the *in vitro* toxicity and cellular uptake of NF loaded with HPPH was assessed, similarly to the NIRFD-containing nanoformulation. As can be expected, the free HPPH exhibited noticeable cytotoxicity, as the cell experiments were performed under ambient light conditions. As a result, the viability of the cultured cells treated with free HPPH was reduced to ∼44.3% ± 4.6% at the highest of the studied concentrations (10 μM). At the same concentration of HPPH but in NF, the cell viability was found to be noticeably higher (71.1% ± 5.0%). For lesser concentrations, the difference between the toxicity of the free and NPs-loaded HPPH is even higher (Fig. 6b). This is apparently associated with the fact that the free HPPH within the cells binds with mitochondria, while the intracellular localization of NPs is not mitochondria-specific.^61,62^

Fig. 6a shows the transmission and fluorescence microscopy images of the LLC cells treated with NP1PS nanoformulation. Similar to the cells treated with NP1D, the cells treated with NP1PS exhibit a fluorescence signal after incubation with NF. Furthermore, the intensity of this fluorescence signal rose by ∼2.5-fold after the cell dish was heated to 40 °C (Fig. 6a and c), revealing that similarly to the cells with NP1D, the NP1PS NF associated with cells manifest its shrinking/aggregating behavior, causing an increase of the fluorescence signal from the post-loaded PS. It should be noted that the fluorescence from cells treated with free HPPH did not noticeably change after heating the cell dish (see ESI, Fig. S5†).

### *In vivo* imaging of nanoformulations with NIRFD and PS

**Fig. 7:**
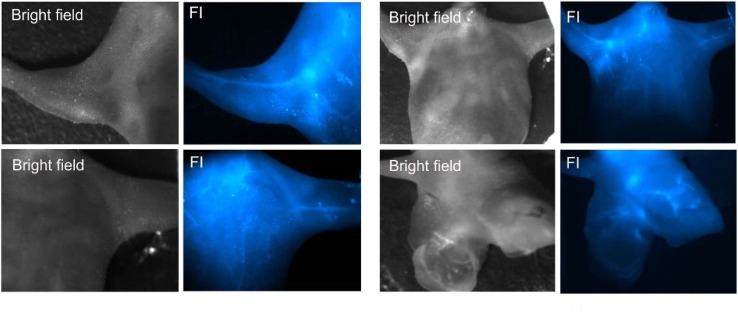
Bright-field and NIR-SWIR fluorescence images of a mouse i.v. injected with NP1D.

After studying the behavior of the fluorescent NF with cultured cells *in vitro*, the experiments on the thermoresponsive fluorescence imaging *in vivo* were performed. We first assessed the injectability of the NF. Fig. 7 shows the NIR-SWIR bright-field and fluorescence imaging of a mouse model intravenously injected with NP1D. The video of the combined NIR-SWIR bright-field/fluorescence imaging of a mouse leg just after i.v. injection of NP1D shows the appearance of the NIR fluorescence in the blood vessels after injection, illustrating the possibility of monitoring the NF distribution in real time (ESI Video SV1,† 4× speed video). Remarkably, the localization of NF changed from organ to organ in the first seconds after i.v. injection, as it can be seen in the NIR-SWIR fluorescence video of the whole mouse body post-injection (ESI Video SV2,† 4× speed video). We hypothesize that this striking redistribution of the NF is associated with the change of the size and properties (*e.g.*, density) of the NPs in the process of their heating from ambient to body temperature after injection.

**Fig. 8:**
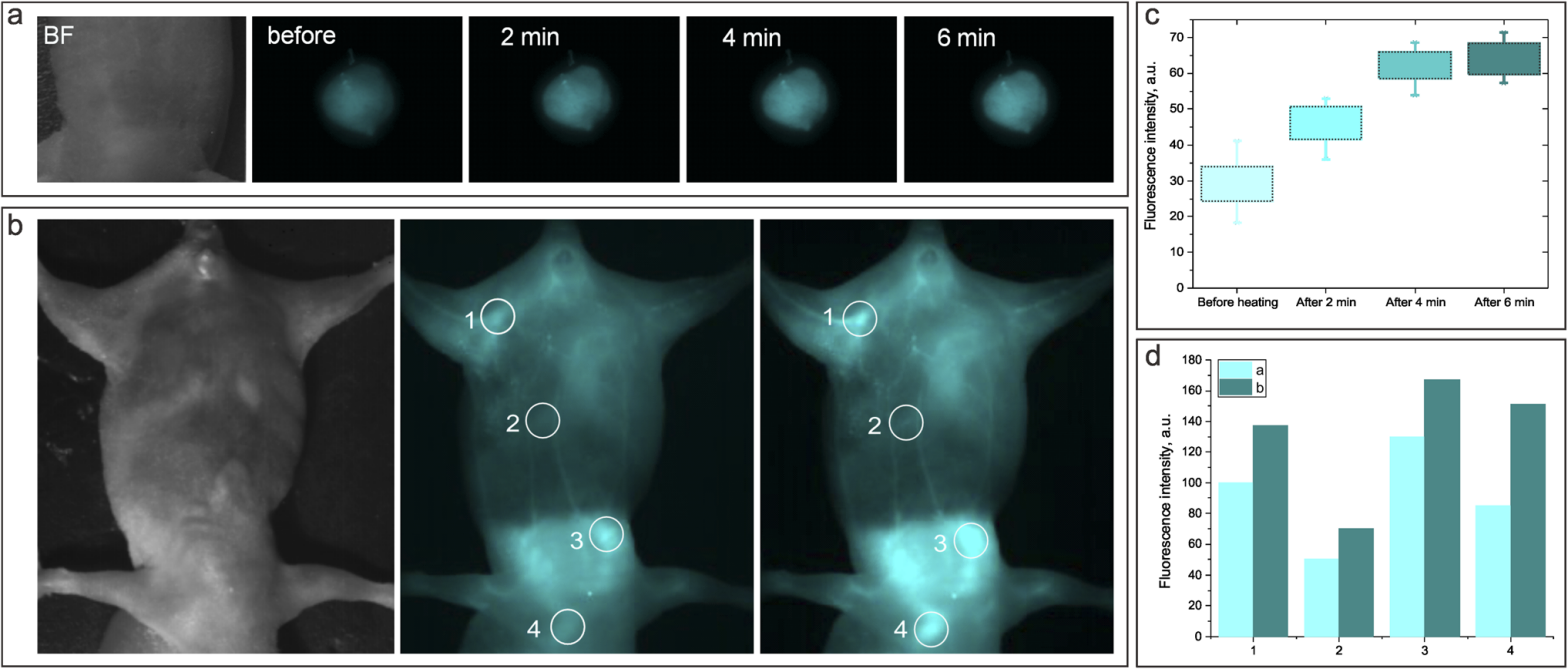
(a and b) Bright-field and NIR fluorescence images of a mouse model subcutaneously (a) and intravenously (b) injected with a NP1D nanoformulation before and after irradiation with a 1300 nm laser; (c and d) histograms of the NIR-SWIR fluorescence intensity from FLI before and after irradiation of the subcutaneous injection site (c) and different locations of the mouse body after intravenous injection of NP1D (d).

Next, we accessed the thermoresponsive behavior of the NIR-SWIR fluorescence from NP1D injected *in vivo* when the specific body location is heated by an external laser source. Fig. 8a shows the NIR-SWIR bright-field and fluorescence images of the mouse's back subcutaneously injected with NP1D NF. It was found that after the injected site was exposed to the 1300 nm laser (with a power density of 50 mW cm^−2^) for 6 min, the NIR-SWIR fluorescence signal from the injected NF increased by ∼2 times as compared to that before laser irradiation (Fig. 8c). Incredibly, the NIR-SWIR fluorescence signal also increased in the regions irradiated by the 1300 nm laser when NP1D NF was injected intravenously (Fig. 8b and d). The NIR-SWIR fluorescence images and fluorescence intensity histograms clearly reveal the increase of the fluorescence signal after 2 minutes of irradiation with the 1300 nm laser.

**Fig. 9:**
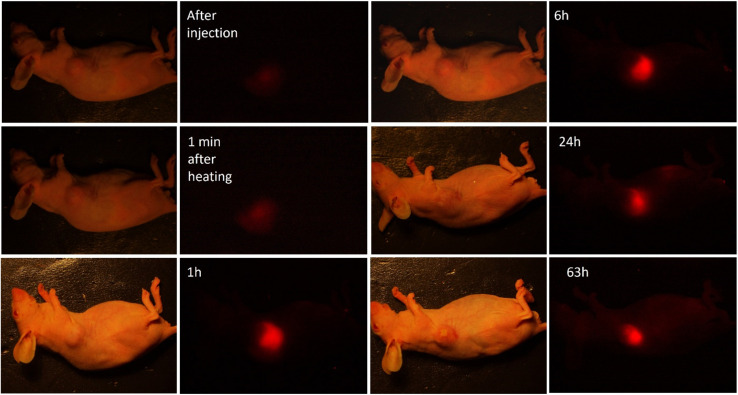
Bright-field and fluorescence images of a mouse tumor model IT injected with NP1PS right after injection, followed by irradiation with a 1300 nm laser. Images were acquired: (1) 1 min after injection, before 1300 nm laser irradiation (heating); (2) 5 min after injection, 1 min after 1300 nm laser irradiation (heating); (3) 1 h, 6 h, 24 h and 63 h post-injection.

Finally, we evaluated the thermoresponsive behavior of the NF containing HPPH. NP1PS was intratumorally injected into LLC tumor-bearing mice, and FLI was performed at certain time points: 1 min after injection; 5 min after injection, and 1 min after 1300 nm irradiation; 1 h; 6 h; 24 h, 63 h. The results of the *in vivo* FLI of are shown in Fig. 9.

The fact that the HPPH fluorescence in the tumor was quite weak after injection of NP1PS, but became noticeably more intense after laser irradiation heating, and was strongly enhanced 1 h post-injection (and heating) can be associated with the initial stage (Fig. 9). It should be noted that the PDT effect depends on the PS concentration in the targeted malignant tissue, which can be quantified by the PS fluorescence.^63^ On the other hand, the quenching of HPPH fluorescence in the previously reported formulations normally correlates with the decrease in singlet oxygen generation and PDT efficiency.^64^ Based on the results of fluorescence spectroscopy and singlet oxygen measurements with NF-containing HPPH (Fig. 5), we suggest that the heating-induced enhancement of the PS fluorescence signal from the mouse tumor injected with NP1PS NF correlates with the increase in the singlet oxygen generation within the tumor, which is beneficial for PDT. The obtained results allow us to assume that the thermoresponsive nanoformulations containing PS can be a promising direction in the imaging guided PDT of cancer, providing a possibility to enhance the fluorescence emission and singlet oxygen production in the diseased tissue selectively heated/irradiated by the NIR laser.

## Conclusions

The developed nanoformulation comprises core–shell nanoparticles with a 30 nm-sized polystyrene core and thermosensitive shell of poly(NIPAM-*co*-AA) co-polymer loaded with fluorescent molecules. The poly(NIPAM-*co*-AA) copolymer in the NPs shell was found to have a LCST value of ∼39 °C, causing a reversible shrinkage of the nanoparticles from the initial (room temperature) size of ∼250 nm down to 160–170 nm for the temperatures above LCST, and further down to ∼140 nm at the highest of the assessed temperatures (∼58–68 °C). To obtain NF, polymethine cyanine dye 3782SL fluorescing at ∼900–1200 nm or PDT agent HPPH, which exhibits fluorescence at ∼660–760 nm, were post-loaded into the NPs shell. While the NIR-SWIR fluorescence of 3782SL and HPPH is quenched in water, it is recovered in the nanoparticle aqueous dispersions as a result of the adsorption of the molecules by the nanoparticle shell. The fluorescence intensity for 3782SL and HPPH in NPs was found to increase with the increase in temperature of the NF above LCST. The rise of the fluorescence intensity was more pronounced for higher NPs concentration. Heating of the HPPH-loaded NFs was found to result not only in an increase in the HPPH fluorescence intensity, but also in the elongation of the fluorescence lifetime and increased singlet oxygen generation. This occurs as a result of the NPs shrinkage at temperatures above LCST, and the corresponding shell compaction and NPs aggregation, which lead to a decrease in the internal conversion rate for fluorescent molecules adsorbed by NPs and a corresponding increase in other electronic excitation deactivation pathways; namely, fluorescence emission and intersystem crossing. The latter, in turn, leads to an increase in the triplet state excitation and corresponding enhancement of singlet oxygen generation. NIR-SWIR fluorescence microscopy revealed a 2-3-fold increase in the 3782SL or HPPH fluorescence signal from the NF-treated cells *in vitro* after they were heated up to 40 °C. Comparable results were obtained for the *in vivo* FLI of mice subcutaneously, intravenously or intratumorally injected with NF. The developed thermosensitive NF holds strong promise for thermally enhanced NIR-SWIR FLI and imaging-guided photodynamic therapy. We believe that the ability to externally control the dynamics of the electronic excitation of the organic molecules loaded to thermosensitive nanoparticles can be of interest for other applications.

## Materials and methods

### Materials

Styrene (St, Ukraine) of p.a. quality was purified *via* standard method directly before polymerization. *N*-Isopropylacrylamide (NIPAM) and *N*,*N*′-methylenebisacrylamide (BIS), both from Sigma-Aldrich (Saint Louis, MO, USA) Inc.), potassium persulfate K_2_S_2_O_8_ and sodium phosphate monobasic dihydrate NaH_2_PO_4_·2H_2_O (both from KPS, Ukraine), were of reagent grade and used without further purification. HPPH (2-[1-hexyloxyethyl]-2-devinyl pyropheophorbide-a) was purchased from Raybiotech (China). Phosphate-buffered saline with pH 7.4 (PBS) was purchased from Gibco Life Technologies (AG, Switzerland). Anionic surfactant sodium dodecyl sulfate (SDS), chloroform, and dimethyl sulfoxide (DMSO) were purchased from Sigma-Aldrich (Saint Louis, MO, USA). Polymethine cyanine dye 3782SL was kindly provided by Dr Yurii Slominskii from the Institute of Organic Chemistry, National Academy of Sciences of Ukraine.

### Synthesis and characterization of thermoresponsive core–shell NPs

Polymeric nanoparticles with a polystyrene (polySt) core and *N*-isopropylacrylamide (NIPAM) and acrylamide (AA) co-polymer shell (polySt-poly(NIPAM-*co*-AA NPs), were synthesized by the modified method of microemulsion polymerization described previously.^40^ In brief, the synthetic protocol was as follows. First, polystyrene core nanoparticles with 10 wt% content of PNIPAM were prepared. Briefly, 0.1 g of NIPAM, 0.1 g of SDS and 0.005 g of NaH_2_PO_4_·2H_2_O were dissolved in 45 mL of H_2_O. Then, the temperature was raised to 50 °C, 1 g of styrene was added dropwise for a duration of 30 min with vigorous stirring (1200 rpm), and Ar was bubbled into the mixture for 30 min. After the temperature reached 70 °C, 0.08 g of K_2_S_2_O_8_ dissolved in 5 mL of H_2_O was injected to initiate the polymerization. Second, the poly(NIPAM-*co*-AA) shell was layered onto the polySt core. For this purpose, monomers of NIPAM (1.63 g) and AA (0.17 g) and the cross-linker *N*,*N*′-methylene bisacrylamide (BIS) (0.18 g) were solubilized in 4 mL of water. The solution was then added into the reaction using a syringe. The reaction was allowed to continue for 2 hours at 70 °C. The mixture was cooled to room temperature, and dialyzed for 72 hours using cellulose membrane with MWCO 3500 Da. As a result, the dispersion of the polySt-poly(NIPAM-*co*-AA) NPs was fabricated.

The TEM imaging of the polySt-poly(NIPAM-*co*-AA) nanoparticles was performed using the HT 7700 (Hitachi Ltd, Tokyo, Japan) transmission electron microscope. The TEM samples were prepared as follows: 10 μL of NPs dispersion with an added contrast agent (phosphotungstic acid, 2 wt%) was dropped onto a carbon support film stabilized with formvar. The size of the NPs was also determined by the dynamic light scattering technique (DLS) with a particle size analyzer (Zetasizer Nano ZS, Malvern). During DLS measurements, the temperature of the samples was changed both ways: from 18 °C to 68 °C (heating) and from 68 °C to 18 °C (cooling) with 2 °C step; every point was acquired after 5 min (2 min pause for the sample stabilization and 3 min for the measurement).

### Preparation and characterization of nanoformulations comprising core–shell NPs and post-loaded fluorophores

To obtain NFs for NIR-SWIR imaging and PDT, NPs were post-loaded with NIRFD or HPPH. In the first step, 10 μL of the NIRFD (3782SL) or PS (HPPH) stock solutions (1 mM in DMF) were added to 90 μL of NPs water dispersion (2% w/v). The resulting dispersions were carefully mixed by micropipette and kept overnight in darkness at room temperature. In the second step, the sample volume was increased to 2 mL by adding water. After careful mixing, the NF were yielded, containing 5 μM of HPPH or 3782SL and 0.1% w/v of NPs. To obtain NF with 1% or 2% w/v of NPs dispersion, the same concentration of HPPH and NIRFD were mixed with 900 μL or 1800 μL of NPs water dispersion, respectively, and then the volume was increased to 2 mL by adding water.

Samples of 0.1%; 1%; 2% w/v NPs in water dispersion were named as NP01, NP1, NP2, respectively. Furthermore, NP01, NP1, and NP2 post-loaded with 3782SL dye were termed as NP01D, NP1D, NP2D, while NP01 and NP1 post-loaded with HPPH were termed as NP01PS and NP1PS, respectively.

The absorption spectra of the dispersions were acquired using a spectrophotometer LAMBDA 750 UV/VIS/NIR (PerkinElmer). Fluorescence spectra in the visible and NIR ranges were obtained using a HORIBA Fluorolog-3 spectrofluorometer coupled for the NIR-SWIR range with an iHR320 spectrometer equipped by the HORIBA DSS-IGA010L point detector. To excite the NIRFD fluorescence, a collimated beam from the fiber-coupled laser diode emitting at 808 nm (QSP-808-4, QPhotonics) was introduced inside the sample chamber of the Fluorolog-3 spectrofluorometer, and directed onto the cuvette with samples of NIRFD solutions or NFs dispersions.

Singlet oxygen generation was evaluated directly *via* its luminescence (phosphorescence) emission peaked at 1270 nm.^41,65^ A Fluorolog-3 spectrofluorometer equipped with an infrared spectrometer (iHR320, Horiba) was employed to detect the singlet oxygen emission. Singlet oxygen phosphorescence was detected by the thermoelectrically cooled NIR-PMT detector (H10330B-75, Hamamatsu) of the iHR320 spectrometer set to 1270 nm. Decays of singlet oxygen phosphorescence under pulsed laser excitation were recorded by the Digital Phosphor Oscilloscope (TDS 3034C, Tektronix) coupled to the output of the NIR PMT. The sample dispersions were placed in quartz cuvettes and excited by the nanosecond Nd:YAG pulsed laser at 532 nm (LS-2137, Lotis TII) and 10 Hz repetition rate.

### Fluorescence imaging

A NIR-SWIR camera (Xeva-1.7-320, Xenics, Belgium) equipped with focusing optics (TEC-M55MPW, Computar, USA) was used to image the NIR-SWIR fluorescence signal from NFs with NIRFD (NP01D, NP1D, NP2D) in a cuvette, as well as the *in vitro* and *in vivo* imaging. For excitation of the NIRFD emission, a fiber-coupled laser diode at 808 nm (QSP-808-4, QPhotonics, USA) powered with the laser power source (Laser Source 4308, Arroyo Instruments, USA) was employed. For acquisition of the NIR-SWIR fluorescence images, an 850 nm long pass filter (Edmund Optics, USA) was used. The illumination sources also included an incandescent lamp (for image alignment, focusing, and bright field imaging) and a 1300 nm fiber-coupled laser diode (QFLD-1300, QPhotonics, USA), which was used for heating the NFs *in vitro* and *in vivo*. A hotplate (Benchmark Scientific, USA) was used to perform imaging of heated NFs in cuvettes in cells *in vitro*. The temperature of the NFs samples in cuvettes under irradiation was measured using a thermocouple and digital multimeter UNI-T (UTM1136C, China).

Fluorescence imaging of NFs with PS (NP01PS and NP1PS) was performed using a Nikon DS-FI2 camera. A Nikon Intensilight C-HGFI lamp with a bandpass optical filter (620 ± 10 nm) was used as an excitation source for HPPH fluorescence. The HPPH fluorescence images were acquired using another band-pass filter (700 ± 17.5 nm). Both filters were purchased from Edmund Optics (USA).

### Cell culture and imaging

LLC cells were grown in Advanced DMEM (Life Technologies) cell medium, supplemented with 10% (v/v) fetal calf serum (FBS) (Sigma, St. Louis, MO), 1% w/v glutamax (Life Technologies), and 1% w/v antibiotic antimycotic solution (Sigma) at 37 °C in a humidified atmosphere containing 5% of CO_2_. The passaging was performed once the cells reached ≈ 80%. Treatment with trypsin (1 : 10 v/v in PBS) was used to detach adherent cells. The number of viable cells was counted upon 0.1% w/v trypan blue staining with a Neubauer chamber.

To measure cell viability, LLC cells (10^4^ per well) were cultured in 96-well cell culture plates for 24 h, and then incubated for the next 24 h in the presence of NFs containing HPPH or 3782SL, free NPs, free HPPH or free 3782SL at equivalent concentrations in 100 μL of cell medium in every well. The used concentrations were: (1) 0.01%, 0.05%, 0.1% for NPs; (2) 0.05 μM; 0.25 μM, 0.5 μM for 3782SL; (3) 0.5 μM; 2.5 μM; 5 μM for HPPH. Free HPPH and 3782SL were added to cells from a stock solution in DMF. Cell viability was determined with the Cell Counting Kit-8 (CCK-8, Sigma-Aldrich).^66^ Briefly, the CCK-8 reagent (10 μL) was added to each well and the cells were incubated for 15 min at 37 °C. The cell viability was determined by measuring the absorption at *λ* = 450 nm with the microplate reader RT-6100, Rayto, USA.

For *in vitro* cellular fluorescence imaging, 2 × 10^5^ LLC cells were seeded into glass-bottom 35 mm dishes (MatTek, Ashland, MA). 24 h after seeding, the cell medium was replaced with that containing NF. NFs containing HPPH or 3782SL (NP1PS or NP1D) were added to the cell medium, resulting in final concentrations of 1.5 μM for HPPH or dye and 0.3% w/v for NPs. Cells were incubated for 24 h at 37 °C, washed with PBS and subjected to fluorescence microscopy. Fluorescence images were captured using the Nikon Eclipse Ti–U microscope coupled with a Nikon Digital Sight DS-Fi2 camera (Nikon, Tokyo, Japan) or NIR camera (Xeva-1.7-320, Xenics, Belgium). The effect of heating on the fluorescence signal from cells treated with NP1PS or NP1D was assessed by placing the dish with LLC cells onto a hotplate set to 40 °C for 2 min, followed by immediate cell imaging using a Nikon microscope.

### Animal studies

BALB/c nude mice were obtained from the Guangdong Medical Laboratory Animal Center (Guangdong, China). Animals were kept at aseptic conditions in a small animal facility. Prior to imaging, male mice (6 weeks old, 20 ± 2 g) were anesthetized with 5 wt% chloral hydrate (0.06 mL per gram of mouse weight) by intraperitoneal injection. For *in vivo* FLI, NP1D or NP1PS containing 5 μM of NIRFD or HPPH were suspended in phosphate buffered saline (PBS), and 70 μL of PBS with NF (containing 0.4 mg kg^−1^ HPPH or 0.2 mg kg^−1^ NIRFD) was subcutaneously/intravenously (NP1D) or intratumorally (NP1PS) injected into mice.

The biodistribution/tumor localization of HPPH delivered with the NP1PS nanoformulation was assessed by fluorescence imaging at different post-intratumoral injection time points (0; 5 min; 1 h; 6 h; 24 h; 63 h). Tumors were generated by injection of 10^7^ LLC cells in 50 μL of PBS subcutaneously into the back of mice. The imaging studies were performed when the tumor volumes reached about 100 mm^3^.

## Ethics approval and consent to participate

All animal experiments were conducted in compliance with the criteria of the National Regulation of China for Care and Use of Laboratory Animals, and under supervision of Shenzhen University's Administrative Panel on Laboratory Animal Care.

## Data availability

The data supporting this article have been included as part of the ESI.†

## Author contributions

Study design and conceptualization, T. Y. O., O. C., A. Y.; methodology, O. C., T. Y. O., A. Y., H. X.; L. O. V.; experiments, O. C.; A. Y.; R. Z.; A. G.; O. A. N.; data analysis, O. C., T. Y. O., A. Y.; L. O. V.; writing – original draft, O. C., T. Y. O.; writing – review and editing, T. Y. O., L. O. V., J. L.; supervision, T. Y. O., J. L.; project administration, H. X., T. Y. O., J. L.; funding acquisition, T. Y. O.; J. L. All authors have read and agreed to the published version of the manuscript.

## Conflicts of interest

The authors declare no competing financial interest.

## Supplementary Material

NA-OLF-D4NA00687A-s001
