# Supplementary material for: Polymeric nanoparticles with a thermoresponsive shell loaded with fluorescent molecules allow for thermally enhanced fluorescence imaging and singlet oxygen generation
Source: Nanoscale Adv. 2025 Jan 9;7(7):1946–61. doi: 10.1039/d4na00687a (PMC11809228; doi:10.1039/d4na00687a)
Supplement: NA-007-D4NA00687A-s001 [file NA-007-D4NA00687A-s001.pdf]

Supplementary Information (SI) for Nanoscale Advances.
This journal is © The Royal Society of Chemistry 2025

Polymeric nanoparticles with thermoresponsive shell loaded with fluorescent molecules allow for thermally enhanced fluorescence imaging and singlet oxygen generation

*Oksana Chepurna[1,2]Artem Yakovliev[1], Roman Ziniuk[1], Anna Grebinyk[3], Hao Xu[1], Olena A. Nikolaeva[4], Andrii I. Marynin[5], Liudmyla O. Vretik[4], Junle Qu[1,6], Tymish Y. Ohulchanskyy[1]**

[1] Key Laboratory of Optoelectronic Devices and Systems of Ministry of Education and Guangdong Province, College of Physics and Optoelectronic Engineering, Shenzhen University, Shenzhen, Guangdong, P. R. China

[2] Department of Neurosurgery, Cedars-Sinai Medical Center, Los Angeles, CA, United States

[3] Deutsches Elektronen-Synchrotron DESY, Zeuthen, Germany

[4] Taras Shevchenko National University of Kyiv, Kyiv, Ukraine

[5] National University of Food Technologies, Kyiv, Ukraine

[6] Engineering Research Center of Optical Instrument and System, Ministry of Education, Shanghai Key Lab of Modern Optical System, School of Optical-Electrical and Computer Engineering, University of Shanghai for Science and Technology, Shanghai, P. R. China

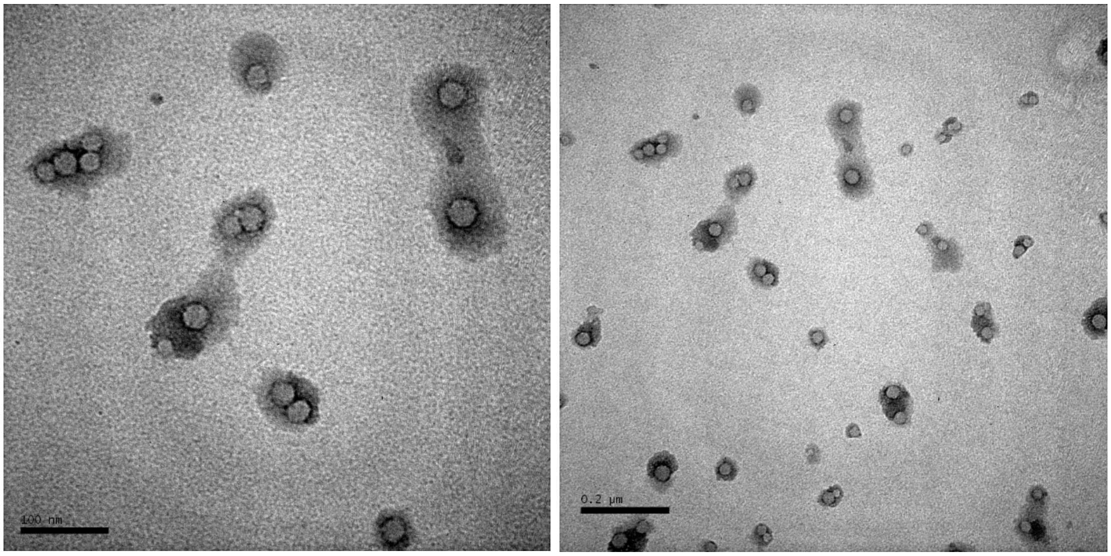

Figure S1. TEM images of polySt-poly(NIPAM-co-AA) core-shell NPs

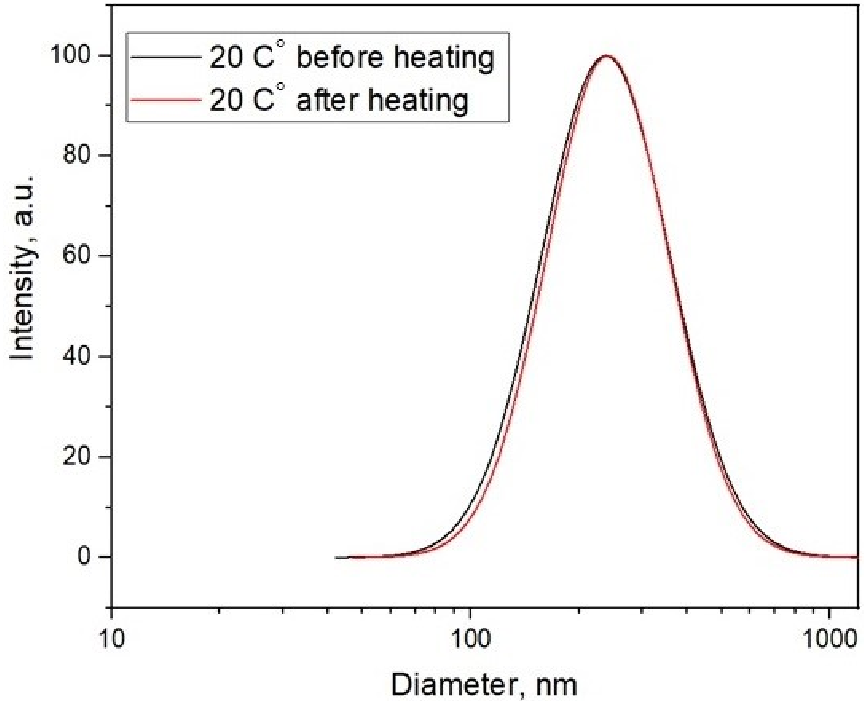

Figure S2. DLS results for NPs water dispersion (0.1 w/v) at 20°C before heating and after cooling.

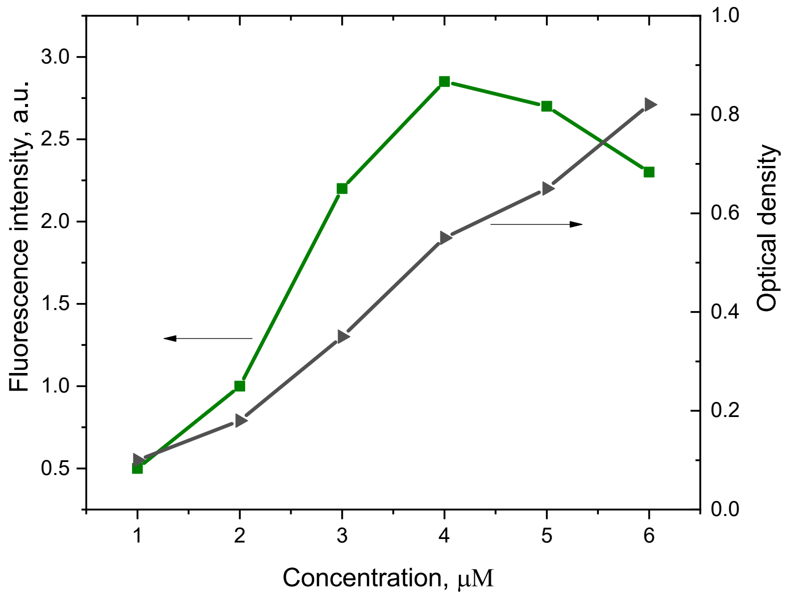

Figure S3. Dependencies of absorption and fluorescence intensity for dye added to NPs dispersion on its concentration.

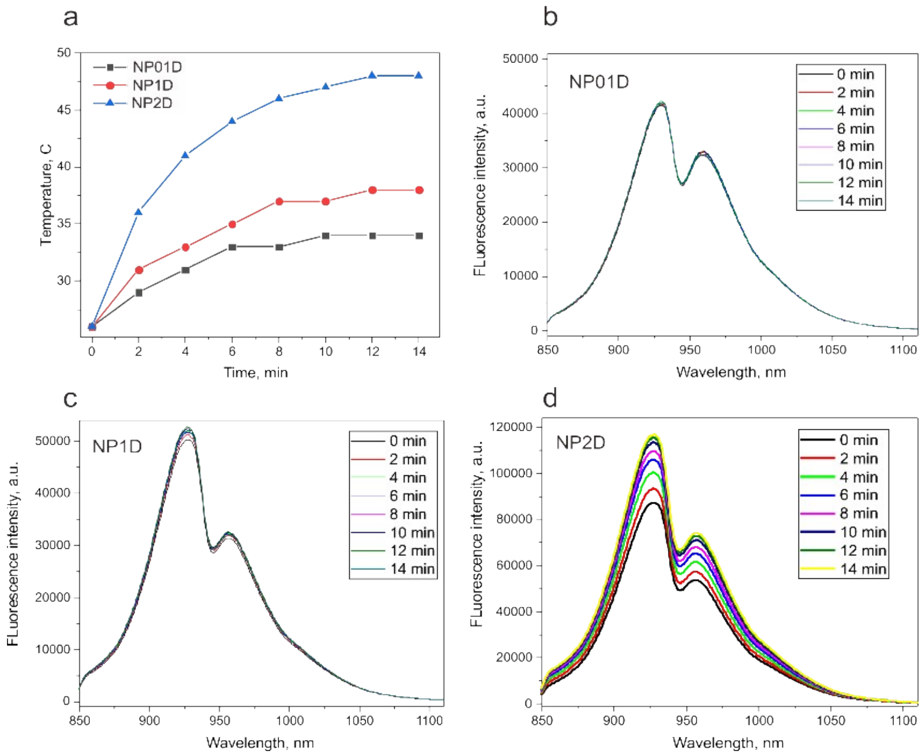

Figure S4. Temperature changes of NFs (NP01D, NP1D, NP2D) under heating with time (0-14 min) (a); fluorescence spectra of NP01D, NP1D, NP2D under heating, excitation at 808 nm (b, c, d respectively).

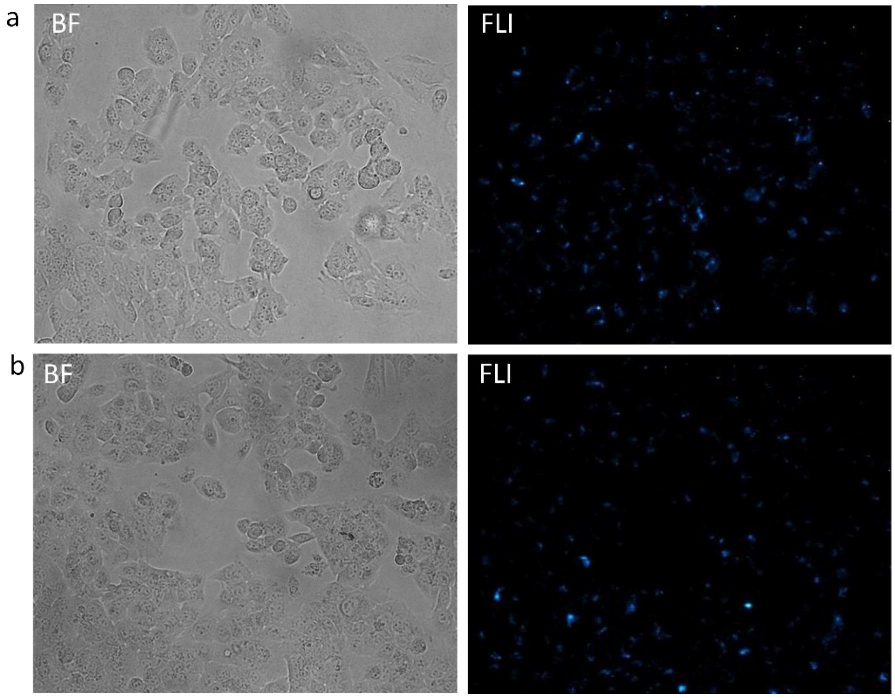

Figure S5. Transmission [BF (bright field), left column] and fluorescence (FLI, right column) microscopy images of LLC cells treated with 3782SL dye, before (A) and after (B) heating.

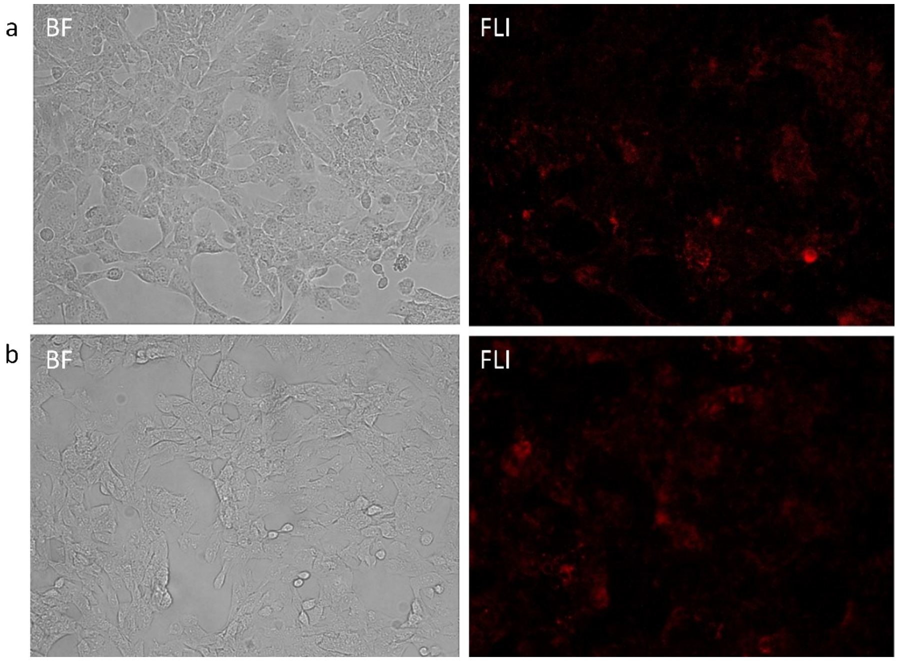

Figure S6. Transmission [BF (bright field), left column] and fluorescence (FLI, right column) microscopy images of LLC cells treated with HPPH, before (A) and after (B) heating.
